# Photosynthetic Membranes of *Synechocystis* or Plants Convert Sunlight to Photocurrent through Different Pathways due to Different Architectures

**DOI:** 10.1371/journal.pone.0122616

**Published:** 2015-04-27

**Authors:** Roy I. Pinhassi, Dan Kallmann, Gadiel Saper, Shirley Larom, Artyom Linkov, Alix Boulouis, Mark-Aurel Schöttler, Ralph Bock, Avner Rothschild, Noam Adir, Gadi Schuster

**Affiliations:** 1 Grand Technion Energy Program, Technion—Israel Institute of Technology, Technion City, Haifa 32000 Israel; 2 Faculty of Biology, Technion—Israel Institute of Technology, Technion City, Haifa 32000 Israel; 3 Schulich Faculty of Chemistry, Technion—Israel Institute of Technology, Technion City, Haifa 32000 Israel; 4 Department of Science and Material Engineering, Technion—Israel Institute of Technology, Technion City, Haifa 32000 Israel; 5 Max-Planck-Institut für molekulare Pflanzenphysiologie, Am Mühlenberg 1, D-14476 Potsdam-Golm, Germany; University of Hyderabad, INDIA

## Abstract

Thylakoid membranes contain the redox active complexes catalyzing the light-dependent reactions of photosynthesis in cyanobacteria, algae and plants. Crude thylakoid membranes or purified photosystems from different organisms have previously been utilized for generation of electrical power and/or fuels. Here we investigate the electron transferability from thylakoid preparations from plants or the cyanobacterium *Synechocystis*. We show that upon illumination, crude *Synechocystis* thylakoids can reduce cytochrome c. In addition, this crude preparation can transfer electrons to a graphite electrode, producing an unmediated photocurrent of 15 μA/cm^2^. Photocurrent could be obtained in the presence of the PSII inhibitor DCMU, indicating that the source of electrons is Q_A_, the primary Photosystem II acceptor. In contrast, thylakoids purified from plants could not reduce cyt c, nor produced a photocurrent in the photocell in the presence of DCMU. The production of significant photocurrent (100 μA/cm^2^) from plant thylakoids required the addition of the soluble electron mediator DCBQ. Furthermore, we demonstrate that use of crude thylakoids from the D1-K238E mutant in *Synechocystis* resulted in improved electron transferability, increasing the direct photocurrent to 35 μA/cm^2^. Applying the analogous mutation to tobacco plants did not achieve an equivalent effect. While electron abstraction from crude thylakoids of cyanobacteria or plants is feasible, we conclude that the site of the abstraction of the electrons from the thylakoids, the architecture of the thylakoid preparations influence the site of the electron abstraction, as well as the transfer pathway to the electrode. This dictates the use of different strategies for production of sustainable electrical current from photosynthetic thylakoid membranes of cyanobacteria or higher plants.

## Introduction

Oxygenic photosynthesis is a sustainable process that converts light energy to fuel products in cyanobacteria, red and green algae and plants [1]. The basic photosynthetic architecture consists of antenna complexes that harvest solar energy and reaction centers that convert the energy into stable separated charge. The photosynthetic apparatus operates through photoexcitation of two linearly connected complexes, photosystem I and II (PSI and PSII) [2,3], coupled by the cytochrome b_6_/f complex (Cytb_6_/f). The initial charge separation occurs in the photosystem II reaction center, the only known natural enzyme that uses solar energy to split water. Both energy transfer and charge separation in photosynthesis are rapid events with high quantum efficiencies. The photosystems are embedded in the thylakoid membranes (hence called thylakoids) that contain the redox complexes responsible for the light-dependent reactions, along with other enzymes and cofactors. In plants and algae the thylakoids are compartmented in the chloroplast, organized into a complex membrane assembly with appressed grana stacks and more open stroma lamella, while in cyanobacteria the membranes are not confined to a subsection of the cell and the do not possess the grana/stroma arrangement. Other architectural differences includes the antenna complexes used by the organisms for light harvesting (the phycobilisome (PBS) in cyanobacteria versus LHCII in plants), the composition of the lipids in the membrane (mostly phospholipids in cyanobacteria versus mostly uncharged galactolipids in plants) and minor peptide compositions [4].

PSII oxidizes water to dioxygen and protons, with the electrons abstracted sequentially by the Oxygen Evolving Complex (OEC). Electrons are transferred in a linear electron transport chain via three electron mediators including the two quinone acceptors, Q_A_ and Q_B_, toward the cytb_6_/f complex [5]. These electrons are eventually used as reducing agent for carbon fixation in the Calvin cycle, while the protons form a gradient which is utilized by the ATP synthase to produce ATP. PSII is composed of 34 intrinsic subunits encoded by *psb* genes, some of which are encoded in the chloroplast in the case of eukaryotic photosynthetic organisms (plants and algae), and the remainder in the nucleus [6]. In the reaction center (RC) of PSII, the integral subunits D1 and D2 (encoded by the *psbA* and *psbD* genes, respectively), bind most of the redox cofactors forming the electron transport chain, assisted by the internal antenna proteins CP47 and CP43, and by the α and β subunits of cytochrome b_559_. Both the D1 and D2 proteins have five transmembrane α-helices (A–E), and two non-transmembrane helices, D-E on the stromal surface and C-D on the lumenal surface [7]. The D-E stromal surface of the D1 protein has been shown to be essential for proper PSII assembly and photoautotrophic growth of the organism; deletions of fragments of this helix in the cyanobacterium *Synechocystis* sp. PCC 6803 (*Syn*) resulted in severe functional perturbations of the Q_B_ electron acceptor of PSII, and prevents autotrophic growth [8].

In recent years, photosynthetic research has been also directed towards the development of synthetic systems that imitate photosynthesis for energy production. Examples include artificial antenna systems capable of funneling excitation energy toward a photosynthesis-inspired reaction centers [9], and the development of inorganic photo-catalysts for water oxidation [10]. Additionally, the potential involvement of photosynthesis in photo-electrochemical solar cells has been investigated through the integration of photosynthetic reaction centers, thylakoids, chloroplasts and whole bacterial cells into hybrid bio-photo-electrochemical devices. For example, PSII reaction centers purified from cyanobacteria were immobilized on a gold electrode surface through cytochromes [11] or through nickel nitrilotriacetic acid as cross linkers [12]. Further, PSII isolated from a thermophilic cyanobacterium was immobilized onto a mesoporous indium–tin oxide (ITO) lattice, and a small photocurrent density (1.6 μA/cm^2^) was documented in the absence of any redox mediator, whereas an enhanced photocurrent density (22 μA/cm^2^) was obtained in the presence of the soluble redox mediator 2,6-dichloro-1,4-benzoquinone (DCBQ) [13]. A bio-photocell composed of a PSII-modified photoanode and a bilirubin oxidase/carbon-nanotube-cathode was shown to generate current upon illumination in the absence of any artificial mediator [14]. Utilization of intact thylakoids membranes has been shown to possess advantages over isolated reaction centers for light harvesting applications such as extended stability towards photochemical damage (photoinhibition, PI), relatively straightforward and non-polluting purification and immobilization methods, and various electron-transfer conduits. Thus, a steady-state current density of 38 μA/cm^2^ was obtained from immobilized spinach thylakoids in a multi-walled carbon nanotubes anode, which was reported to retain activity for a week [15]. A stable photocurrent density of 130 μA/cm^2^ was obtained for few hours from spinach thylakoids immobilized on a modified gold electrode, using the electron mediator *para*-benzoquinone [16].

In this work, we have studied the electrochemical properties and currents produced using crude thylakoids from *Syn* or thylakoids isolated from the higher plants, spinach and tobacco. We compare the abstraction of photosynthetic electrons by the exogenous protein cytochrome c (cyt c), and present a bio-photocell capable of producing photocurrents from illuminated thylakoids. By employing a graphite anode in the photocell we eliminate the necessity to use expensive electrode materials. Additionally, crude thylakoid preparations are used without any immobilization step, though the photocurrents detected in our simple device are quite significant. Moreover, we demonstrate that by genetic engineering of the PSII reaction center the currents obtained from *Syn* thylakoids are significantly enhanced. However, unlike what observed with the thylakoids of *Syn*, electrons cannot be abstracted directly from plant PSII and a mediator is required to obtain a photocurrent. Therefore, different strategies should be applied for using cyanobacterial or plant thylakoids for solar energy conversion.

## Results

### Cyt c is reduced by PSII in *Syn* but not in spinach thylakoids

**Fig 1:**
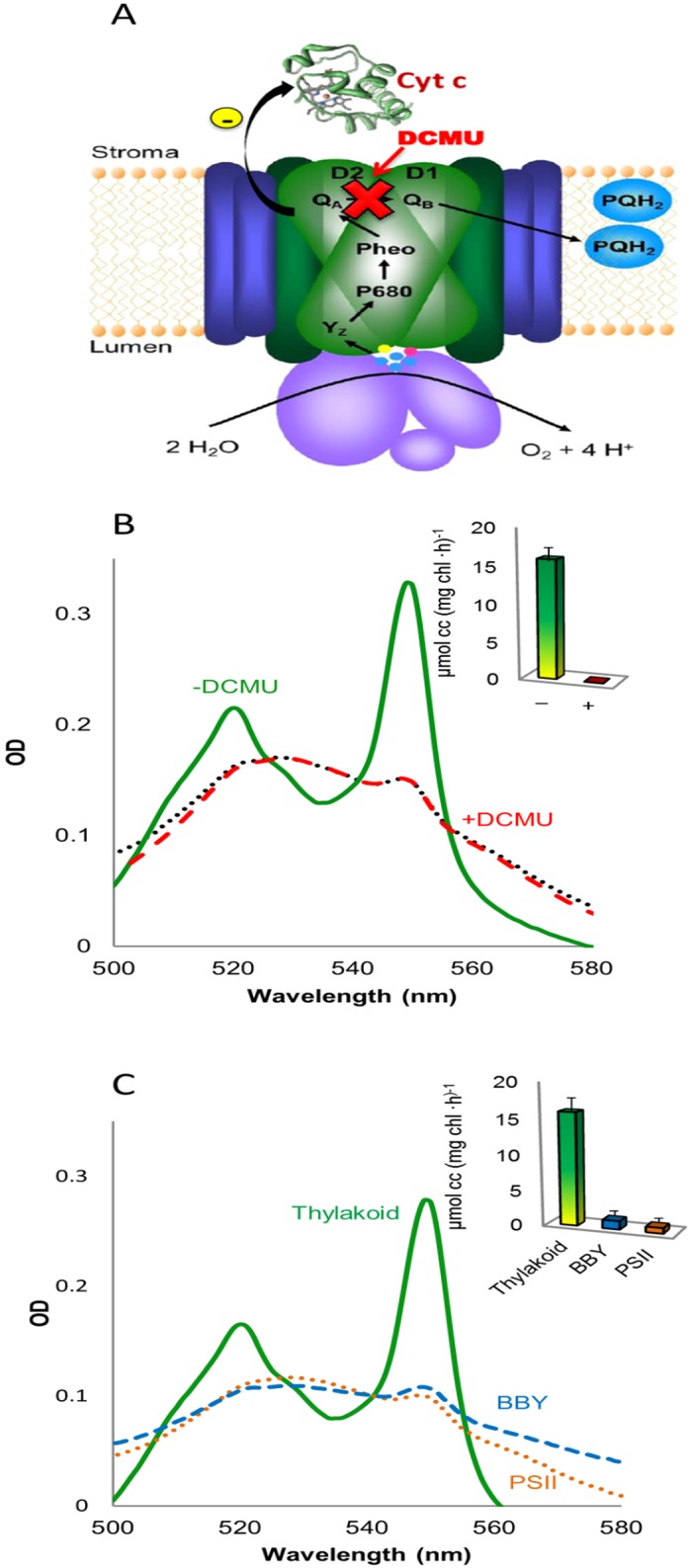
Cyt c is reduced by PSII in *Syn* but not in spinach thylakoids. **A**. Schematic illustration of the postulated electron transfer pathway from PSII in *Syn* thylakoid to cyt c_ox_. Photo-oxidized electrons are postulated to be transferred from Q_A_ to cyt c. The addition of DCMU blocks linear electron flow between the Q_A_ and Q_B_ and thus increases the reduction rate. **B**. Stacked spinach thylakoids were incubated for 3 min with cyt c_ox_ in the dark (black dotted), light (green) or in the light with the addition of DCMU (red dashed). Following centrifugation of the membranes, the absorption spectra of the cyt c containing supernatant was measured. The concentration of reduced cyt c was calculated using the coefficient Δϵ550nm-542nm. The inset depicts the quantification of cyt c photoreduction from three independent experiments in the absence (green) or presence of DCMU (red). **C**. Analysis of cyt c photoreduction at different levels of spinach PSII isolation. Unstacked spinach thylakoids (Thylakoid, solid green), PSII enriched membranes (BBY, dashed blue) or purified PSII (PSII, dotted brown) were incubated and analyzed as described for panel A. The inset depicts the quantification of cyt c photoreduction by the unstacked thylakoids (green bar) PSII enriched membranes (blue bar) or purified PSII (brown bar) from three independent experiments. No photoreduction occurred in the presence of DCMU.

The unique catalytic activity of PSII offers an intriguing platform for the production of sustainable energy by solar energy conversion. A crucial step in the developments of such systems is the ability to abstract electrons from the insulating thylakoids. Thus, abstraction of photosynthetically-derived electrons by cyt c was demonstrated from thylakoids of the *Syn* mutant D1-K238E, and to a lesser extent from wild type (WT) thylakoids [17]. In both cases, the addition of DCMU increased the abstraction rate, implying that electrons were abstracted prior to Q_B_ (most likely from Q_A_), as illustrated schematically in Fig 1A.

We monitored the transfer rates of electrons from spinach (*Spinacia oleracea*) thylakoids to cyt c by visible light spectroscopy, as described in the Experimental section. Fig 1B shows spectra of oxidized cyt c before (cyt c_ox_) and after incubation with spinach thylakoids. Upon the co-incubation, a significant peak at 550 nm is observed, which is indicative of cyt c reduction [18] Quantification of the cyt c reduction rate is presented at the inset of Fig 1B, showing a nearly ten-fold faster reduction rate compared to the values reported for wild-type *Syn* on a chlorophyll basis [17], and ~2.5 fold on the basis of the relative amounts of PSII in these two photosynthetic organisms. To probe the source of the electrons that reduce cyt c, DCMU was added. In wild-type *Syn* thylakoids a five-fold increase in cyt c reduction was obtained in the presence of DCMU, while with spinach thylakoids, DCMU completely inhibited the reduction of cyt c. This indicates that in spinach thylakoids the reduction site is not on the surface of PSII, near Q_A_. To further investigate this observation, PSII-enriched membranes (BBY membranes) were purified [19]. These membrane fragments are almost devoid of all other photosynthetic complexes and despite the enrichment of PSII (indicated by the Chl_a_/Chl_b_ ratio and higher oxygen evolution rate, S1 Table) the BBY membranes showed negligible cyt c reduction capacity, which was completely inhibited by DCMU (Fig 1C). One possibility for the apparent difference between the thylakoids might be due to the localization of most of the active PSII with the appressed grana stacks which are characteristic of plant thylakoids but not cyanobacterial thylakoids. Spinach thylakoids were therefore unstacked by sequential washing in low-salt buffers lacking Mg^+2^ and containing EDTA [20]. Unstacked thylakoids were used throughout the following experiments unless otherwise is indicated. The unstacking procedure did not affect the rate of oxygen evolution, nor affect the cyt c reduction in the absence or presence of DCMU, as compared to the rate of reduction obtained from stacked thylakoids. Lastly, PSII complexes were isolated by solubilization with the detergent β-dodecyl-maltoside. The highly active isolate, as indicated by the increased oxygen evolution rate (S1 Table) was incubated with cyt c in the light. However, no photoreduction in the presence or absence of DCMU could be detected (Fig 1C). The reduction rates of cyt c by the spinach preparations mentioned are presented in the inset of Fig 1C.

### Photocurrent from *Syn* or plant thylakoids is produced differently

**Fig 2:**
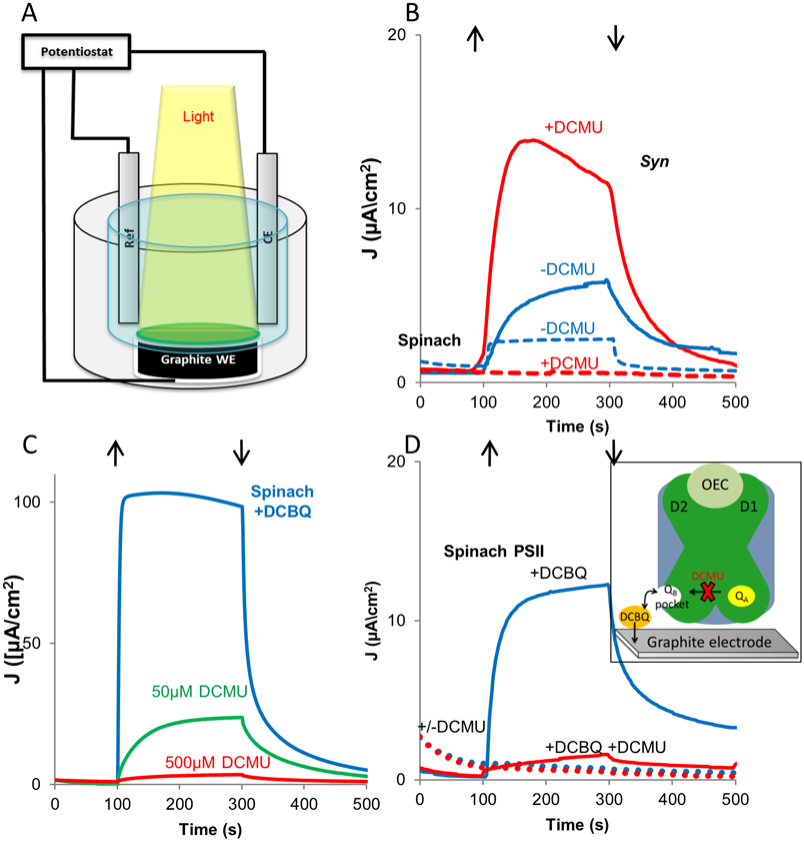
*Syn* thylakoids transfer a direct photocurrent from Q_A_ while higher plants require the presence of an electron transfer mediator. **A**. Schematic illustration of the photocell used for photocurrent measurement. A suspension of thylakoid membranes were allowed to settle onto a graphite anode placed at the bottom of a sealed container in a minimal amount of buffer B. The electrode was illuminated from the top by a xenon lamp calibrated to provide an equivalent spectrum of 1 sun. Currents were measured by a potentiostat connected in 3-electrode mode to an Ag/AgCl reference electrode and to a Pt cathode. **B**. Direct photocurrent produced by wild-type-*Syn* (solid lines) or spinach (dashed lines) thylakoids illuminated on the graphite anode in the absence (blue) or presence (red) of 50 μM DCMU under a bias of 0.05V_Ag/AgCl_. Up and down facing arrows indicate light on and off, respectively. **C**. DCBQ mediated photocurrent produced by spinach thylakoids on the graphite anode in the absence (blue), the presence of 50μM (green) and of 500 μM DCMU (red) under a bias of 0.24 V_Ag/AgCl_. **D**. Photocurrent produced by isolated spinach PSII on the graphite anode with (dotted lines) or without (solid lines) 0.3mM DCBQ, in the absence (blue) or presence (red) of 50 μM DCMU as in panel B. The inset schematically depicts the role of DCBQ as electron mediator. DCBQ_OX_ diffuses into the Q_B_ pocket, and is reduced by Q_A_^-^ (DCBQ_red_). DCBQ_red_ then diffuses to the anode where it is re-oxidized to become available for another electron transfer cycle.

We proceeded to construct a bio-photoelectrochemical cell (BPEC) in which photosynthetic electrons could be transferred to electrodes and monitored as a photocurrent. The schematic of such a cell is shown in Fig 2A. Dark and light currents produced in the photocell by *Syn* thylakoids were monitored under external bias of 0.05V_Ag/AgCl_. In accordance with recently reported data obtained using a modified gold working electrode [21] unmediated electron transfer from the wild-type *Syn* thylakoids to a graphite electrode was obtained, reaching current densities of 6 μA/cm^2^ (Fig 2B). As described above, when DCMU was added to the system, a resulting increase in the photocurrent to 15 μA/cm^2^ was obtained (Fig 2B).

Embracing a more sustainable and economic approach and in order to make use of plants instead of cyanobacteria, we addressed the use of (unstacked) thylakoids purified from spinach to explore electron abstraction in the same photocell configuration. Since spinach thylakoids exhibited improved electron transfer to cyt c, we anticipated the plant thylakoids to produce far greater photocurrent than obtained from *Syn* thylakoids. Unexpectedly, the opposite was observed, and the measured unmediated current density was only 2 μA/cm^2^ (Fig 2B). Addition of DCMU did not enhance current production as for the *Syn* thylakoids but rather completely inhibited it. To see whether indirect electron transfer was possible, the exogenous electron acceptor DCBQ was added to the system. DCBQ is a well characterized redox-active compound possessing reversible electrochemical properties with conductive surfaces [16]. Due to structural resemblance to Q_B_, DCBQ_ox_ diffuses into the Q_B_ pocket, where it is reduced by electrons coming from Q_A_ [22]. In the cell presented here, DCBQ_red_ was anticipated to diffuse toward the electrode, transfer the electron and disengage for another charge transport cycle. In this configuration, an external bias of 0.24V_Ag/AgCl_ was applied, and a photocurrent density of 100 μA/cm^2^ was obtained (Fig 2C). Since DCBQ accepts electrons downstream to Q_A_, co-incubation of spinach thylakoids with DCMU was anticipated to hinder the DCBQ induced photocurrent. As seen in Fig 2C, a competitive effect governs the process: at 50μM of DCMU the photocurrent was inhibited by about 60%, while 500μM DCMU entirely blocked the photocurrent. Finally, to verify that PSII was the electron source, PSII purified from spinach in β-dodecyl-maltoside [23,24], was analyzed, as presented in Fig 2D. As with intact thylakoids, a direct photocurrent could not be detected in the presence or absence of DCMU, indicating that PSII purified from spinach did not transfer electrons directly to the graphite. However, electron transfer from PSII was detected following the addition of DCBQ, which was again inhibited by DCMU. We note that while the electron transfer pathway obtained from isolated PSII or from spinach thylakoids may be identical, the photocurrent densities differ, which demonstrates that the connectivity of membranes and electrode plays an important role, and is architecture/preparation dependent. The proposed electron transfer pathways from spinach thylakoid is illustrated in the inset of Fig 2D.

### The Glu mutation does not improve electron transfer from tobacco PSII

Larom *et al*. reported that genetic manipulation of amino acid 238 of the D1 PSII protein of *Syn* from lysine to glutamate (K238E) caused a significant increase in the reduction of cyt c [17], which increased even further in the presence of DCMU. Lysine 238 of D1 is situated in the D-E loop, an extra-membrane hood-like structure, posed above Q_A_. Since all our attempts to abstract electrons from plant PSII were unsuccessful and the positive amino acid at position 238 of D1 is evolutionarily conserved in all photosynthetic organisms [17], we asked whether the homologous mutation in higher plants would have the same effect in opening of a pathway for the abstraction of electrons to cyt c or the graphite anode, as obtained in *Syn*. The biolistic transformation technique was used on tobacco plants for genetic manipulation of the chloroplast genome, where the protein D1 is encoded by the *psbA* gene. Instead of a lysine, higher plant D1 contains an arginine at position 238 of the D1 protein. This arginine was modified to either alanine (t-R238A), aspartate (t-R238D) or glutamate (t-R238E), in an attempt to create a range of chemical modifications in the D-E loop vicinity (S1 Fig). Selection of these mutations was facilitated by the concomitant introduction of an *aadA* cassette conferring resistance to spectinomycin and streptomycin [25]. A line carrying only the *aadA* cassette and the wild type *psbA* sequence (WT-*aadA*) was produced to serve as a control. Since there are many copies of the chloroplast genome in each chloroplast and there are multiple chloroplasts in each cell, it was important to select homoplasmic lines in which all DNA molecules of the chloroplast genomes are identical and harbor the genetic modification. Homoplasmy was achieved by conducting multiple rounds of regeneration under stringent antibiotic selection and was confirmed by restriction fragment length polymorphism (RFLP) analysis by Southern blotting (S2 Fig) followed by sequencing of the *psbA* gene in the chloroplast genome of the transformed (transplastomic) plants (S3 Fig). Several homoplasmic lines for each mutation were selected, from which plants were regenerated and seeds collected. Two of these independent lines were then used to grow the plants used throughout this study, and RFLP analysis (S2 Fig) and DNA sequencing (S3 Fig) were employed to verify the correct sequence of the mutated *psbA*.

Plants harboring the genetic modifications in the chloroplast genome grew autotrophically, with no visible phenotype (Table 1 and S4 Fig). Furthermore, biochemical and spectroscopic methods were used to compare common photosynthetic parameters of the tobacco lines including oxygen evolution rate, maximum linear electron transfer rate / linear electron transport capacity (ETRII), chlorophyll content and a/b ratio, and the contents of major photosynthetic complexes: PSI, PSII and the cytb_6_/f. Table 1 compares the phenotypes and photosynthetic parameters of wild type (WT) tobacco to lines R238E and WT-*aadA*. No statistically significant differences between the tobacco lines were observed. Comparison between all the tobacco lines produced and the wild type is presented in the S2 Table and S4 Fig. As previously observed for the identical mutations in the *Syn psbA* gene, there were no significant differences in the photosynthetic characteristics of the tobacco mutants.

**Table 1:** Photosynthetic parameters of the D1-R238E tobacco mutant.

**Parameter**	**Units**	**WT**	**WT-*aadA***	**R238E**
O_2_ evolution rate*	(μmol O_2_/(mg Chl ·h))	123±6	122±8	118±6
Leaf absorbance	(%)	87.6±0.8	87.8±1.2	87.0±1.3
Fv/Fm	N/A	0.8	0.8	0.8
ETR^§^	(μmol e^-^/m^2^/s)	133±11	131±10	117±6
Chl content^§^	(mg/m^2^)	535±20	531±61	501±59
Chl_a_/Chl_b_ ratio		4.0±0.1	4.0±0.1	3.9±0.1
PSI content^§^	(μmol/m^2^)	1.3±0.1	1.3±0.2	1.2±0.1
b_6_f complex^§^	(μmol/m^2^)	0.6±0.1	0.6±0.1	0.5±0.1
PSII content^§^	(μmol/m^2^)	1.3±0.1	1.5±0.3	1.3±0.2

Thylakoids of mutated tobacco lines in which arginine 238 of the D1 protein was modified to glutamate (R238E) display similar photosynthetic characteristics to thylakoids of WT and WT-*aadA* tobacco lines.

*O_2_ evolution was determined with a Clark type electrode, with DCBQ as the exogenous electron acceptor.

^§^The maximum light-saturated linear electron transport capability (ETR), chlorophyll (Chl) content and *a/b* ratio and the content of major photosynthetic complexes photosystem I (PSI), photosystem II (PSII), and the cytochrome b_6_f were determined spectroscopically, as detailed in the Experimental section. Values are average of six independent experiments, and presented ± standard error.

**Fig 3:**
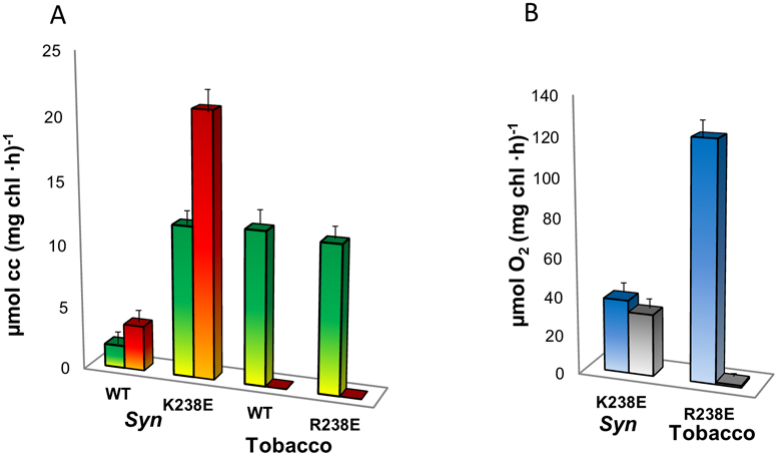
Modification of the conserved amino acid at position 238 of the D1 protein increased electron abstraction from Q_A_ in *Syn* but not in tobacco. **A.** Quantification of cyt c (cc) photoreduction by thylakoids of WT-*aadA* tobacco, WT-*Syn*, *Syn* D1-K238E or tobacco D1-R238E. In the absence (green) or following the addition of the herbicide DCMU (red). DCMU enhanced electron transfer by *Syn* thylakoids, but completely blocked electron transfer in tobacco. Data was collected in three independent experiments. Bars represent standard error. **B.** Oxygen evolution rates monitored as a measure of the light-dependent electron transfer from the PSII oxygen evolving center of mutated *Syn* and tobacco (K238E *Syn*, R238E tobacco, respectively). The exogenous electron acceptors used were DCBQ in the absence of DCMU (blue bars) or cyt c in the presence of the herbicide DCMU (grey bars).

Cyt c reduction was compared between thylakoids of the wt and modified lines of *Syn* and tobacco, as presented in Fig 3A. While, as reported previously, a four-fold faster cyt c reduction was obtained with thylakoids purified from the K238E strain of *Syn* as compared to WT- *Syn*, [17] no difference in cyt c reduction was observed when comparing the tobacco lines R238E and WT-*aadA*. Moreover, while the addition of the herbicide DCMU enhanced cyt c reduction in the K238E strain of *Syn*, it completely inhibited cyt c reduction in tobacco thylakoids. Since there was the possibility that the lack of observable reduced cyt c was due to fast reoxidation by an unknown tobacco thylakoid component, we performed complementary oxygen evolution measurements using cyt c as the exogenous acceptor. Oxygen evolution rates of 120 and 40 μmol O_2_ (mg chl ·h)^-1^ were obtained from thylakoids of the mutated tobacco and *Syn* thylakoids, respectively, using DCBQ as the exogenous acceptor (Fig 3B). When cyt c was used as the exogenous electron acceptor in the presence of DCMU, the rate of oxygen evolution produced by the mutated *Syn* thylakoids was close to the level obtained with DCBQ, indicating a similar electron flow from O_2_ to DCBQ throughout PSII and Q_B_ and from O_2_ to cyt c via Q_A_. However, no oxygen evolution could be detected in the presence of DCMU when the WT-*aadA* and mutated tobacco thylakoids where analyzed, indicating that the electrons were obtained downstream of the Q_B_ site (Fig 3B).

### A mediator is required in order to produce electricity using tobacco thylakoids

**Fig 4:**
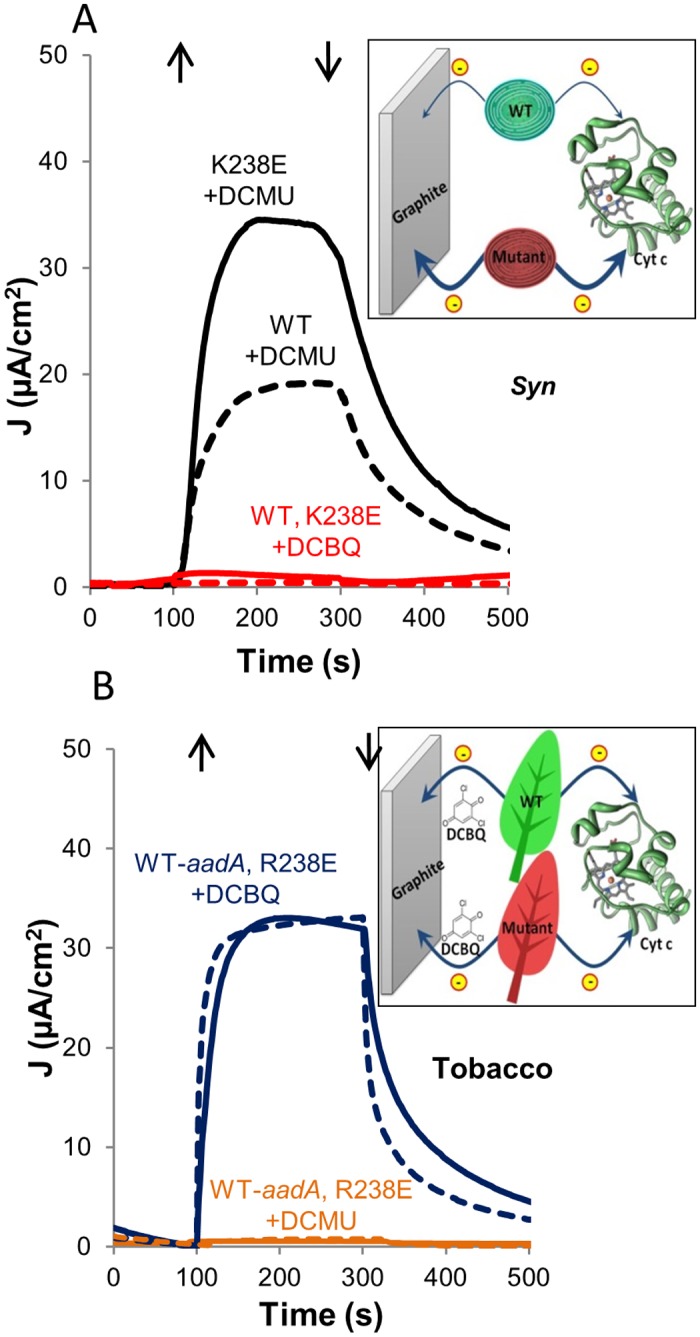
A mediator is required in order to produce photocurrent with tobacco thylakoids. **A.** *Syn* thylakoids transfer electrons directly to the electrode (black, no mediator added). The addition of DCBQ (red) resulted in inhibition of the photocurrent. Direct and DCBQ-mediated photocurrents produced by thylakoid of WT (dashed lines) or K238E (solid lines) strains of *Syn* were measured on a graphite-working electrode under external bias of 0.05V_Ag/AgCl_ and 0.24V_Ag/AgCl_, respectively. The inset illustrates the schematic representation of the electron abstraction pathways from the *Syn* thylakoids. Thin and thick arrows indicate the magnitude of electron transfer, respectively. **B.** Unmediated (orange) and DCBQ-mediated (blue) photocurrent obtained from WT-tobacco (dashed lines) or R238E-tobacco (full lines) as described in panel A. Inset illustrated the electron abstraction pathways from the tobacco lines.

In order to analyze the potential of the thylakoids to produce a significant electric current, thylakoids were placed in the photocell described above (Fig 2A) and electric currents were monitored. When thylakoids of the wild-type or mutated K238E strain of *Syn* were analyzed in the photocell, a direct photocurrent was obtained in the presence of DCMU (Fig 4A). Furthermore, the photocurrent densities obtained from the mutated strain were higher compared to the WT reaching a photocurrent density of 35 μA/cm^2^. The higher current obtained with the K238E is in a good correlation to the increased cyt c reduction observed with this strain compared to WT (Fig 3 panels A, B). Interestingly, when we investigated whether the direct electron transfer pathway could be augmented or replaced by a DCBQ-mediated one, we found that the photocurrent decreased to nearly zero (Fig 4A). In Fig 4A all components were present prior to measuring current. When we initiated the measurement only in the presence of DCBQ, no current was obtained (S5 Fig). However, addition of DCMU at this later stage indeed rescued the direct electron transfer pathway (S5 Fig). We believe that this indicates that while DCBQ inhibits the indirect pathway (in *Syn*), addition of DCMU blocks DCBQ reduction, allowing direct electron transfer to the graphite anode.

The introduction of a mutation in the D1 protein of tobacco did not affect DCBQ mediated electron transfer from the thylakoids to the graphite anode (Fig 4B). Accordingly, in both the mutated R238E and the WT-*aadA* control lines similar DCBQ-mediated photocurrents were monitored and DCMU inhibited the photocurrent (Fig 4B). No significant differences in the mediated photocurrent between the tobacco lines were observed, indicating that the mutation did not affect electron transferability. As opposed to the situation in *Syn*, no direct photocurrent could be observed with DCMU in higher plants thylakoids, both in the WT or mutated strains. The insets in Fig 4 panels A and B summarize the electron transfer patterns observed.

## Discussion

Thylakoids accommodate many biochemical functionalities performed by different complexes, depending on species. The most important components are the four multi-subunit photosynthetic protein complexes which are assembled into highly organized systems, warranting a substantial degree of stability. At the same time, they exhibit remarkable structural flexibility, which plays important roles in short-term adaptation mechanisms in response to changing environmental conditions, including extremities of temperature, irradiance and salinity [26]. The catalytic water splitting activity of PSII which is responsible for the linear electron flow and protons release offers a platform for the sustainable production of electricity and/or the production of fuels through green chemistry. Inherently, thylakoids are insulating material, and thus the inaccessibility of photosynthetically derived electrons is a hurdle which must be overcome. We used thylakoids purified from cyanobacteria or plants and investigated approaches to collect these electrons. First, we showed that electrons could be abstracted from thylakoids of *Syn* or tobacco by the redox active protein cyt c, however the pathways through which the electrons were collected differed between the organisms. In *Syn* the source of abstraction is probably Q_A_, while in plants this site appears to be shielded, so electrons are abstracted after PSII, possibly from the cytochrome b_6_/f complex or from PSI.

Next, we constructed a photocell which produced a photocurrent of about 15–100 μA/cm^2^ from *Syn* or spinach thylakoids. However, as explained for the reduction of cyt c, the sites of electron abstraction differed. Additionally, we showed that significant mediator-free photocurrents from *Syn* thylakoid were obtained, while a mediator was needed when using plant thylakoids. We note that mediator-free coupling of the photosystem to an electric circuit is favorable from kinetic and thermodynamic perspectives; mediated electron transport is rate-limited by diffusion, and the electron potential is that of the mediator. Hence, the bias required for the photocurrent production in the mediated pathway was higher. The higher energetic input may also be attributed to the cathode reaction; we postulate that using *Syn* in an unmediated manner the external bias supports hydrogen evolution at the cathode, while in the mediated manner a cyclic electron transfer to DCBQ_ox_ dominates the cathode reaction. Further work to clarify the cathode reactions is now underway.

Further, we examined the recently reported correlation between the ability of mutagenized thylakoids to reduce cyt c and the ability to transfer electrons to an electrode in a photocell using [21] tobacco as a model for plants. Based on the high similarity of the pigments, co-factors and proteins between cyanobacteria and plants we hypothesized that the introduction of the same mutation into plant PSII would produce a similar effect, thereby potentially providing large amounts of relatively easily grown stock material for a bio-solar energy conversion device. To this end, we observed that the incorporation of this mutation to tobacco failed to open a conduit from PSII to either cyt c (or to introduce a cyt c binding site of the appropriate affinity) or to the graphite anode in the photocell, thereby validating a negative correlation between the inability of mutagenized thylakoids to reduce cyt c and the inability to transfer electrons to an electrode. These findings raise the question why *Syn* and plant thylakoids differ with respect to electron abstraction from PSII. Since the tobacco thylakoids were unstacked (and thus similar to cyanobacterial thylakoids), we suggest that the observed difference between the cyanobacterial and plant thylakoids may be due to one or more peripheral PSII components, possibly as the consequence of the procedure applied to purify the thylakoids. For example, the architecture of the Q_A_ vicinity in isolated thylakoids, which in vivo is shielded by the peripheral antenna systems attached to the membrane, PBS or LHCII in cyanobacteria or plant thylakoid, respectively, probably differ between the organisms as a result of the detachment of the PBS during the isolation procedure. This may open an electron channel from Q_A_ and provide an access for the cyt c reduction, as well as for electron transfer to the graphite electrode. Moreover, the enhancement in stability of the Q_A_^-^ species in the presence of DCMU explains the increased electron transferability from *Syn* thylakoids in its presence. Since LHCII remains intact during thylakoid isolation from plants, a similar electron conduit does not open from the vicinity of Q_A_. [4]. Future investigation of electron abstraction from *Prochlorophytes*, a group of cyanobacteria which do not use the PBS for light-harvesting, but instead contain an intrinsic Chl-binding proteins resembling the LHCII [27], may shed more light whether or not this possibility accounts for the observed differences.

Another possibility relies on the methods employed for thylakoid purification; a mild shockwave is used to break cyanobacterial cells during the preparation, whereas a more forceful sheering and grinding action is used to break leaf tissues. It may be case that the aggressive sheering step applied on plant leaves perforates the lumen, thus prevents its acidification during the photosynthetic activity. The inability to build up a proton motive force on the lumen membrane in purified plant thylakoids may hinder the electron abstraction observed in *Syn*.

Alternatively, electron abstraction from PSII in *Syn* may depend on the existence of conductive pili, which has been reported in some cyanobacteria, including *Syn* [28]. These nanowire structures have been shown to facilitate extracellular electron transport from the cells to distant extracellular electron acceptors in a way resembling the abstraction of electrons described here. It has been argued that such objects may serve to funnel excess photosynthetic electrons when they cannot be consumed for carbon fixation, to prevent the accumulation of harmful oxidative stress in the reaction center. If so, the modification of D1 position 238 in *Syn* may have affected electron transfer through this natural pathway. Experiments to monitor photocurrent from *Syn* thylakoids with enriched or depleted pili structures are underway. Controlling the expression of such apparent nanowire-type objects is an exciting approach to increase photocurrents in the future.

## Materials and Methods

### Plant material and bacterial strains

*Synechocystis* sp. PCC 6803 wild-type and mutated strains were grown in BG-11 medium under white light (50 μE/m^-2^·sec^-1^) at 27°C^12^. Spinach (*Spinacia oleracea*) was produced by the Strauss Company Ltd. and purchased at local supermarkets. The spinach was grown either in open fields or in greenhouses in the Eastern Galilee between the following coordinates: North: 33°4'18.4" to South 32°51'50.5", East 35°16'32.8" to West 35°6'24.2". Tobacco plants (*Nicotiana tabacum* cv Petit Havana) used for genetic transformation were grown under aseptic conditions on agar-solidified Murashige and Skoog (MS) medium containing 30 g/L sucrose [29]. Transplastomic lines were rooted and propagated on the same medium. Plants for analysis and seed production were grown in soil under standard greenhouse conditions. Inheritance and seedling phenotypes were analyzed by germination of surface-sterilized seeds on MS medium containing 500 mg/L spectinomycin.

### Generation of genetically modified tobacco plants

#### Construction of the CrPrrn-T7g10-aadA-Cr3’rbcL

The *aadA* coding sequence was amplified by PCR (primers: AadA-NdeI_Flast and AadA-XbaI_R; template: plasmid pRB8) [30]. The PCR product and the vector pDK131 (containing the 5’P*rrn* and 3’*rbcL* sequences of *Chlamydomonas reinhardtii*, obtained by inserting the *Sac*I-*Hind*III-digested fragment of pDK133 [31] into a pBS-KS backbone) were digested by *Nde*I and *Xba*I, and the appropriate products purified on gel, then ligated and transformed into competent *Escherichia coli* TOP10F’ cells by heat shock. The *Cr*P*rrn*-*aadA*-*Cr*3’*rbcL* sequence was verified by sequencing. The cassette was excised by digestion with *Ecl*136II and *Cla*I and treated with the Klenow fragment of *E*. *coli* DNA polymerase I to obtain blunt ends.

#### Construction of the pAB vectors

A two-step PCR procedure [32] allowed the insertion of the mutations in the tobacco *psbA* sequence: two pairs of primers, psbA-BamHI_F2/mut_R and mut_F/rpl2-EagI_R1 (where mut corresponds to R238A, R238D and R238E for the pAB13, pAB14 and pAB15 plasmids, respectively), allowed amplification from wild-type DNA of two partially overlapping fragments, which were mixed and used as templates in a third PCR with the external primers psbA-BamHI_F2 and rpl2-EagI_R1. The final amplicons, each of them carrying one mutation, were digested by *Eco*RI and *Eag*I and cloned into plasmid pBS-SK^-^ digested with the same enzymes. The *Cr*P*rrn*-*aadA*-*Cr*3’*rbcL* cassette was then inserted into the *Spe*I site filled-in by the Klenow fragment, downstream and in direct orientation with respect to the *psbA* sequence. The sequences of the oligonucleotides used are listed in S3 Table.

#### Plastid transformation and selection of homoplasmic transplastomic tobacco lines

Young leaves from aseptically grown tobacco plants were bombarded with plasmid-coated 0.6 μm gold particles using a PDS1000He Biolistic gun (Bio-Rad). Primary spectinomycin-resistant lines were selected on regeneration medium containing 500 mg/L spectinomycin [25]. Spontaneous spectinomycin-resistant plants were eliminated by double selection on medium containing spectinomycin and streptomycin (500 mg/L each). For each transformation construct, several independent transplastomic lines were subjected to two to three additional rounds of regeneration on spectinomycin-containing medium to enrich the transplastome and select for homoplasmic tissue. Physical maps of the *psbA* gene area of the wild type (WT) and the transgenic (R238A, R238D, R238E, WT-aadA) tobacco lines are drawn in S5 Fig.

### Isolation of nucleic acids and hybridization procedures

Total plant DNA was isolated by a cetyltrimethylammoniumbromide-based method [33]. DNA samples were digested with the restriction enzymes BsmI, BseRI or EcoRI that cut at the modified loci of *psbA* in lines R238A, R238D and R238E, respectively, separated on 0.8% agarose gels, and blotted onto Hybond N nylon membranes (GE Healthcare). For hybridization, [α-^32^P]dATP-labeled probes were generated by random priming (Multiprime DNA labeling kit; GE Healthcare). Restriction fragments covering the *psbA* gene were used as probes for the restriction fragment length polymorphism (RFLP) analyses in transformed plants. Hybridizations were performed at 65°C in rapid hybridization buffer (GE Healthcare) following the manufacturer's protocol.

### Chlorophyll-a fluorescence measurements

Chlorophyll-*a* fluorescence of intact leaves was measured at 22°C using a Dual-PAM-100 instrument (Heinz Walz GmbH, Effeltrich, Germany). The maximum quantum efficiency of PSII photochemistry (F_V_/F_M_) and light-response curves of linear electron transport (ETRII) were measured on intact leaves after at least 30 min of dark adaptation. To calculate linear electron transport rates (ETRII), for each actinic light intensity, the PSII operating efficiency was multiplied with the corresponding photosynthetically active photon flux density, assuming an equal distribution of excitation energy between the two photosystems. The linear electron transport rates were corrected for the leaf absorbance measured with an integrating sphere (ISV-469, Jasco) attached to the Jasco V-550 spectrophotometer. Transmittance and reflectance spectra of leaves were recorded between 400 and 700 nm wavelength, and leaf absorbance was calculated as 100% minus transmittance of light through the leaf minus reflectance on the leaf surface. The average value of the absorbance spectrum between 400 and 700 nm was used for the calculation of linear electron flux, assuming an equal distribution of absorbed light between both photosystems.

### Thylakoid membrane and PSII isolation

The procedure for thylakoid membranes preparation from *Synechocystis* cyanobacteria was described by Komeda et al. [34]. Isolations of crude thylakoid preparation from spinach and tobacco were conducted according to Anderson et al. [35]. Unstacking of grana lamella was performed by sequential washing of crude thylakoids in Mg^+2^ deficient buffers supplemented with Ethylenediaminetetraacetic acid (EDTA, Sigma) as described by Dekker et al. [20]. Grana stacks enriched thylakoid preparations, namely BBY membranes, were extracted using the detergent Triton-X (Sigma) described by Berthold et al. [19]. Highly active photosystem II was purified from spinach or tobacco using the detergent n-octyl-glycoside (Anatrace), followed by solubilization of the isolate in micelles of the detergent n-Dodecyl-β-D-Maltoside (Thermo Scientific) as previously published [23,24]. The chlorophyll content in thylakoid preparations was determined according to Arnon [36]. Oxygen evolution activity of the preparations was determined using a Clark-type electrode (Hansatech Instruments, UK) with 10 μg chl of the isolated thylakoid preparations in buffer B (50mM MES/NaOH pH = 6.0, 15mM NaCl, 5mM MgCl, 2mM CaCl_2_) and 0.3 mM 2,6-dichlorobenzoquinone (DCBQ, Sigma) as the exogenous electron acceptor. The suspension was incubated for 5 min at 25°C in the dark, and then illuminated with white light for 1 min followed by a 1 min dark interval, three times per sample, and the increase in oxygen concentration was measured digitally. The rate of oxygen evolution was calibrated according to the manufacturer's instructions with solutions bubbled with either N_2_ (containing 0% O_2_) or air.

### Difference absorption spectroscopy

The contents of PSII, the cytochrome b_6_f complex, and PSI were determined in thylakoids isolated according to Schöttler et al. [37]. PSI was quantified from P700 difference absorption signals at 830 to 870 nm wavelength in solubilized thylakoids using the Dual-PAM instrument (Waltz, Germany). PSII and the cytochrome b_6_f complex were determined from difference absorbance measurements of cytochrome b_559_ (PSII) and cytochromes f and b_6_. Thylakoids equivalent to 50 μg chlorophyll ml^−1^ were incubated in a low-salt medium to improve the optical properties of the sample by unstacking the thylakoids. All cytochromes were oxidized by application of 1 mM sodium ferricyanide. Addition of 10 mM sodium ascorbate resulted in the reduction of cytochrome f and the high-potential form of cytochrome b_559_, while cytochrome b_6_ and the low-potential form of cytochrome b_559_ were only reduced upon addition of dithionite. At each redox potential, absorption spectra between 575- and 540-nm wavelengths were determined using the V-550 spectrophotometer (Jasco, USA) with a head-on photomultiplier. The monochromator slit width was set to 1 nm. The difference absorption spectra were deconvoluted using reference spectra and difference absorption coefficients as described by Kirchhoff et al. [38]. The PSII content was calculated from the sum of the difference absorption signals arising from the low and high potential forms of cytochrome b_559_.

### Cytochrome c reduction

5 μg chl of isolated thylakoid preparations in buffer B were incubated with 25 μM horse heart cyt c (Sigma-Aldrich) in the dark with or without 50 μM 3,4-dichlorophenyl-1,1-dimethylurea (DCMU, Sigma), and illuminated for 3–5 min with white light. The concentration of reduced cyt c was calculated using the coefficient Δϵ550–542 = 21mM^-1^ cm^-1^, obtained by a standard calibration curve.

### Electrochemical measurements

Photocurrents produced by isolated thylakoid preparations (1 mg Chl/ml), BBY membranes (1 mg Chl/ml) or solubilized PSII (0.15 mg Chl/ml) were analyzed in a designated photocell illustrated in Fig 2D at 25 ±2°C. 150 μl of isolated thylakoids preparations in buffer B, added with 10% v/v glycerol to allow easy settle of the suspension, were set on a graphite sheet working electrode placed at the bottom of the photocell, and top illuminated by a xenon lamp calibrated to provide spectra of 1 sun (Abet Technologies, Inc., USA). The operating conditions for electrochemical tests were chosen based on a series of cyclic voltammetry measurements, and conducted by a potentiostat (Zahner-Elektrik, Germany) connected in a 3-electrode mode cell setup with a Ag/AgCl (3M KCl) reference electrode (CH Instruments) and a platinum wire counter. Direct and DCBQ-mediated (0.3 mM) photocurrents were measured by chronoamperometry, under external bias of 0.05 V_Ag/AgCl_ and 0.24 V_Ag/AgCl_, respectively, under alternating light and dark condition with different concentration of DCMU.

## Supporting Information

S1 Fig Physical maps of the *psbA gene* area of the wild type (WT) and the transgenic (R238A, R238D, R238E, WT-*aadA*) tobacco lines. (TIF)

S2 Fig Restriction fragment length polymorphism (RFLP) analysis was conducted to confirm homoplasmy. (TIF)

S3 Fig Growth phenotypes of wild type tobacco (WT) and the transgenic lines WT-*aadA* (control), R238A, R238D and R238E. (TIF)

S4 Fig The *psbA* of tobacco plants used for cyt c reduction and electrochemical analysis were amplified by PCR and sequenced to confirm the genomic sequence. (TIF)

S5 Fig A DCBQ-mediated electron transfer from *Syn* thylakoids could not be established. (TIF)

S1 Table PSII activity at different isolation levels. (DOCX)

S2 Table Photosynthetic parameters of tobacco mutants. (DOCX)

S3 Table List of oligonucleotides used for the generation of the mutated tobacco lines. (DOCX)
