# Supplementary figures and images for: Photosynthetic Membranes of Synechocystis or Plants Convert Sunlight to Photocurrent through Different Pathways due to Different Architectures
Source: PLoS One. 2015 Apr 27;10(4):e0122616. doi: 10.1371/journal.pone.0122616 (PMC4411099; doi:10.1371/journal.pone.0122616)

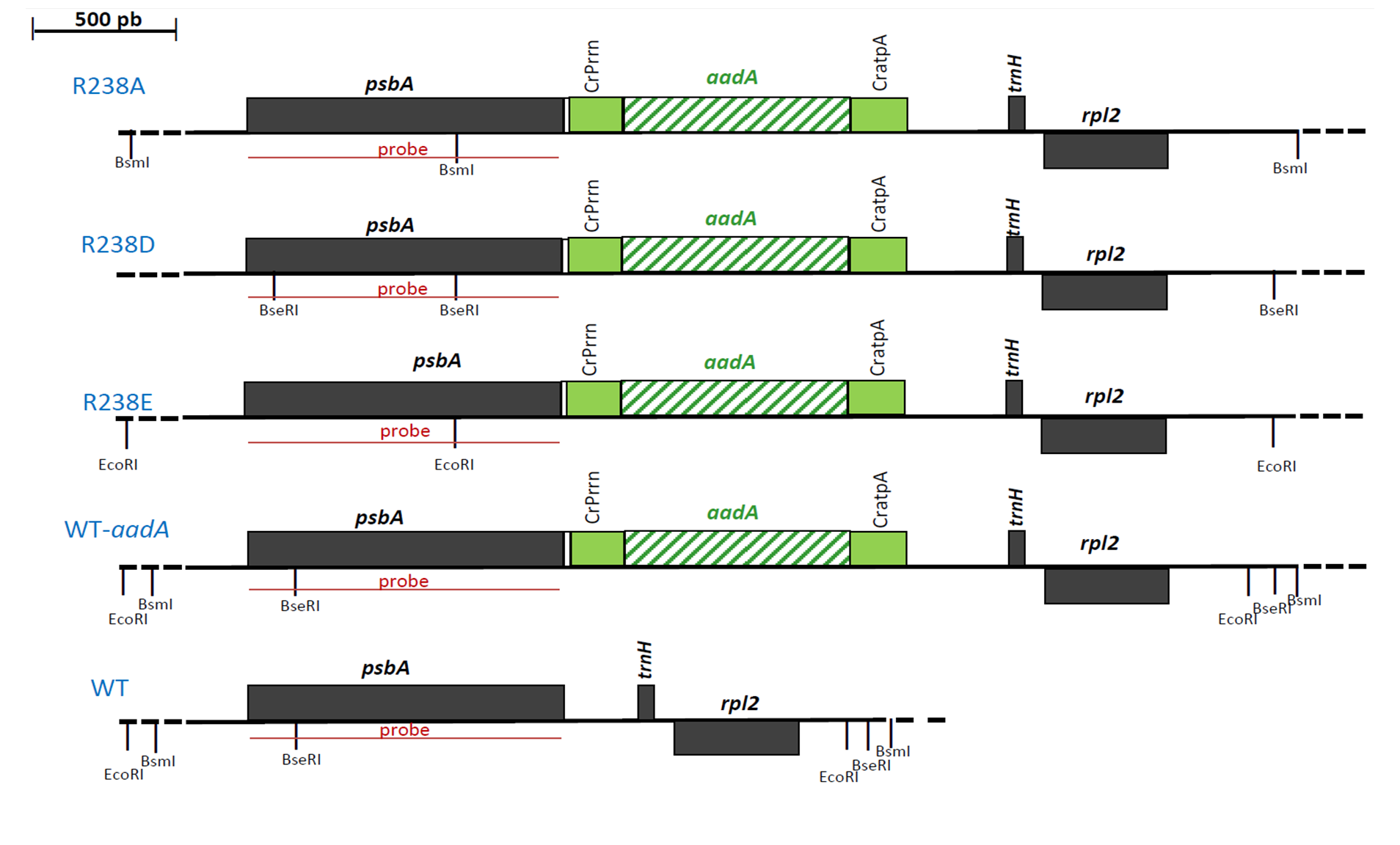

Supplement: S1 Fig — (TIF) [file pone.0122616.s001.TIF]

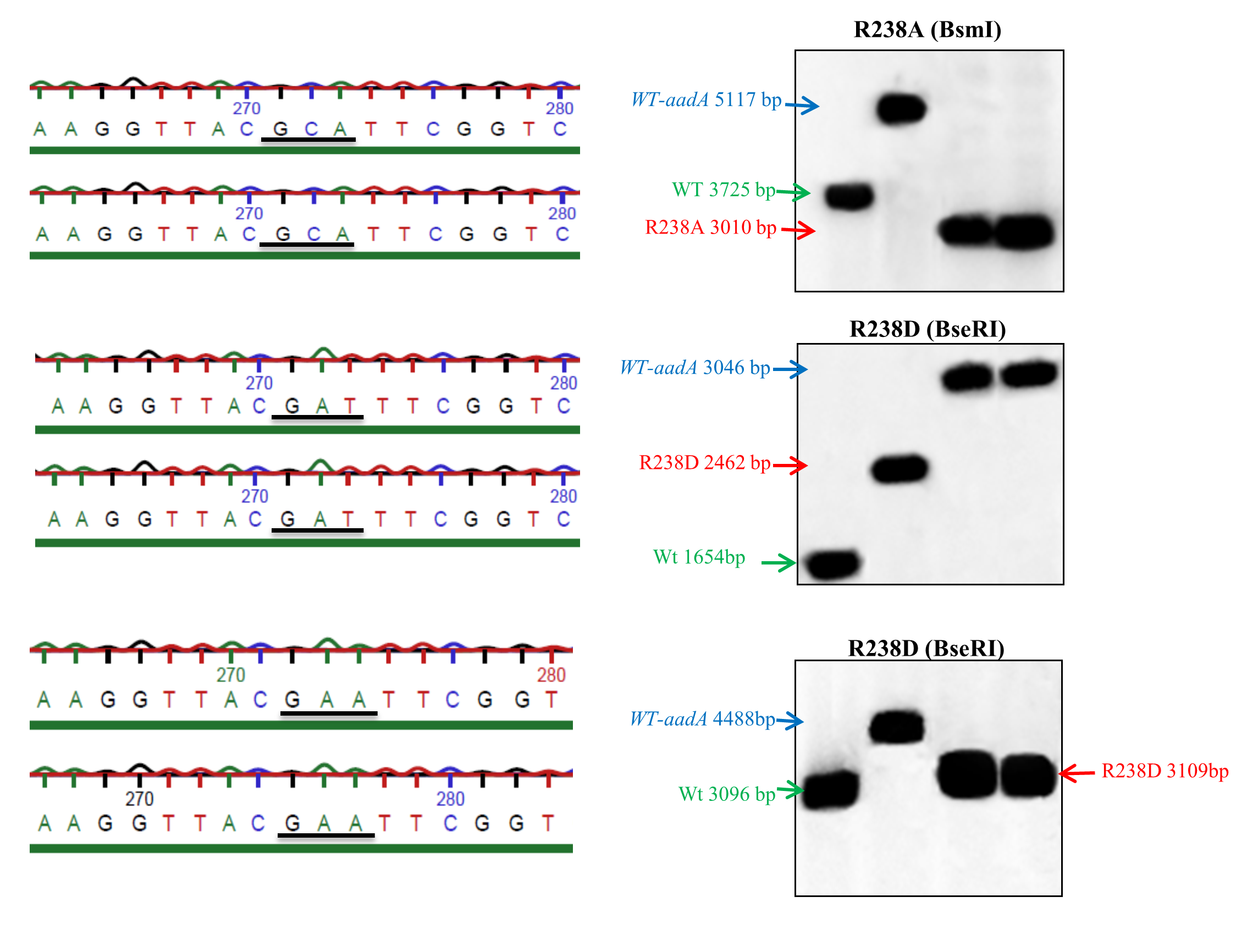

Supplement: S2 Fig — (TIF) [file pone.0122616.s002.TIF]

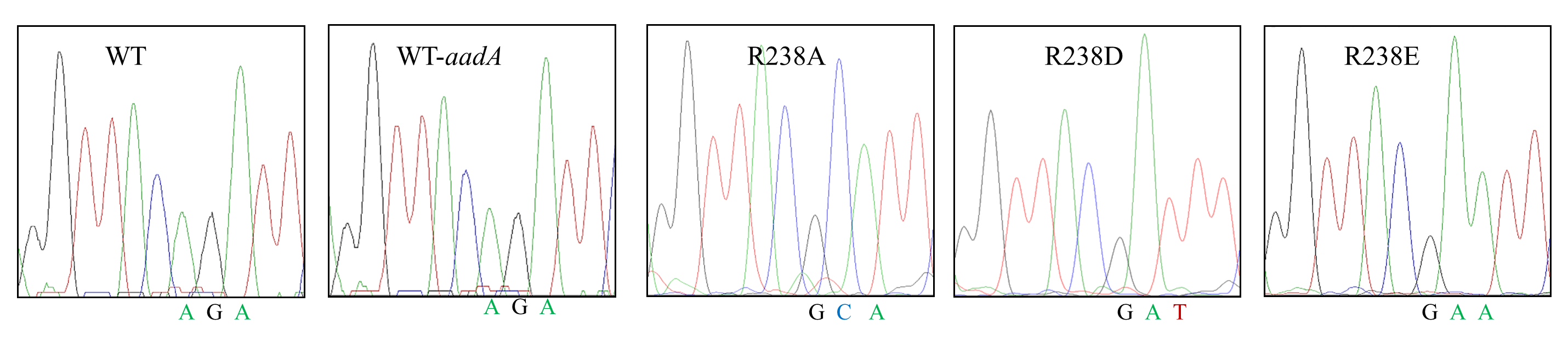

Supplement: S3 Fig — (TIF) [file pone.0122616.s003.TIF]

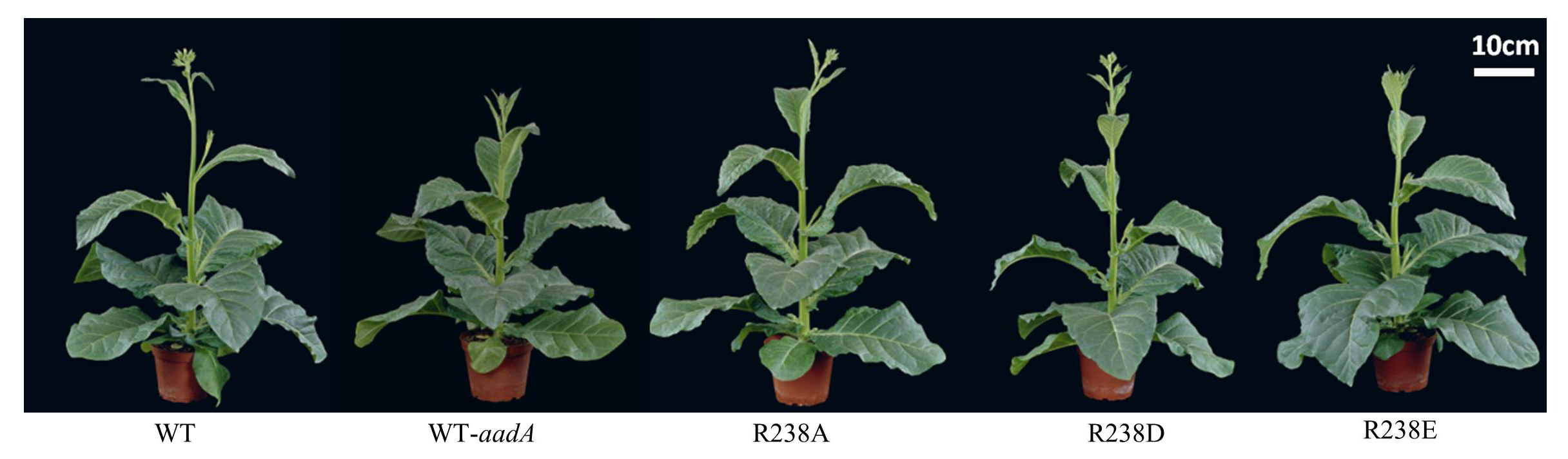

Supplement: S4 Fig — (TIF) [file pone.0122616.s004.tif]

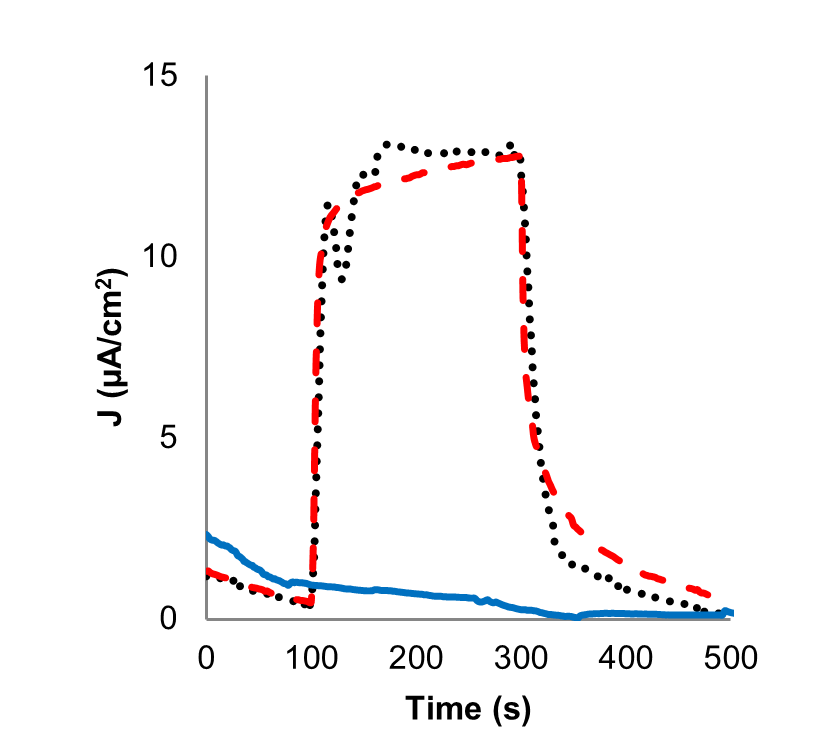

Supplement: S5 Fig — (TIF) [file pone.0122616.s005.TIF]
