# Supplementary material for: Photosynthetic Membranes of Synechocystis or Plants Convert Sunlight to Photocurrent through Different Pathways due to Different Architectures
Source: PLoS One. 2015 Apr 27;10(4):e0122616. doi: 10.1371/journal.pone.0122616 (PMC4411099; doi:10.1371/journal.pone.0122616)
Supplement: S1 Table — (DOCX) [file pone.0122616.s006.docx]

**Table S1. PSII activity at different isolation levels.** Oxygen evolution rates produced by crude thylakoid membranes, BBY membranes and solubilized PSII in β-DM from spinach and the tobacco lines WT, WT-*aadA*, R238D or R238E. Values are the average of four independent experiments, and presented ± standard errors.

| **Preparation** | | **WT spinach** | **WT tobacco** | **WT-*aadA* tobacco** | **R238E tobacco** |
| --- | --- | --- | --- | --- | --- |
| **Crude thylakoids** |  | 210±12 | 123±6 | 122±8 | 118±6 |
| **BBY** |  | 360±20 | 188±15 | 196±12 | 179±14 |
| **Solubilized PSII** |  | 620±18 | 320±10 | 348±16 | 306±10 |
