# Supplementary material for: Photosynthetic Membranes of Synechocystis or Plants Convert Sunlight to Photocurrent through Different Pathways due to Different Architectures
Source: PLoS One. 2015 Apr 27;10(4):e0122616. doi: 10.1371/journal.pone.0122616 (PMC4411099; doi:10.1371/journal.pone.0122616)
Supplement: S2 Table — (DOCX) [file pone.0122616.s007.docx]

**Table S2. Photosynthetic paramters of tobacco mutants.** Thylakoids of mutated tobacco lines, in which arginine 238 of the D1 protein was mutated to either alanine (R238A), aspartate (R238D) or glutamate (R238E) were characterized by ten different photosynthetic parameters. All strains displayed similar photosynthetic characteristics and cyt-c reduction rates as thylakoids of WT and WT-*aadA* tobacco lines

| **Parameter** | **Units** | **WT** | **WT-*aadA*** | **R238A** | **R238D** | **R238E** |
| --- | --- | --- | --- | --- | --- | --- |
| **O_2_ evolution rate*** | (µmol O_2_*mg Chl*hr^-1^) | 123±6 | 122±8 | 121±7 | 128±6 | 118±6 |
| **Leaf absorbance** | (%) | 87.6±0.8 | 87.8±1.2 | 87.3±1.8 | 88.2±1.2 | 87.0±1.3 |
| **Fv/Fm** |  | 0.8 | 0.8 | 0.8 | 0.8 | 0. 8 |
| **ETR^§^** | (µmol e^-^*m^-2^*sec^-1^) | 133±11 | 131±10 | 120±22 | 110±5 | 117±6 |
| **Chl content^§^** | (mg*m^-2^) | 535±20 | 531±61 | 514±76 | 530±20 | 501±59 |
| **Chl_a_/Chl_b_ ratio^§^** | - | 4.0±0.1 | 4.0±0.1 | 3.9±0.1 | 3.9±0.1 | 3.9±0.1 |
| **PSI content^§^** | (µmol*m^-2^) | 1.3±0.1 | 1.3±0.2 | 1.3±0.2 | 1.3±0.1 | 1.2±0.1 |
| **b_6_f complex^§^** | (µmol*m^-2^) | 0.6±0.1 | 0.6±0.1 | 0.6±0.1 | 0.6±0.1 | 0.5±0.1 |
| **PSII content^§^** | (µmol*m^-2^) | 1.3±0.1 | 1.5±0.3 | 1.4±0.1 | 1.3±0.1 | 1.3±0.2 |
| **Cyt c reduction rate^§^** | (µmol cc*mg Chl^-2^*h^-2^) | 12±1 | 12±1 | 11±2 | 13±1 | 12±1 |

*O_2_ evolution was determined by Clark type electrode, with DCBQ as the exogenous electron acceptor. **^§^**Linear electron transfer rate (ETR), cyt-c reduction rate, Chlorophyll (Chl) content and ratio and the content of major photosynthetic complexes photosystem I (PSI), photosystem II (PSII), and the cytochrome b_6_f were determined spectroscopically, as detailed in the Methods. Values are average of six independent experiments, and presented ± standard errors.
