# Supplementary material for: Photosynthetic Membranes of Synechocystis or Plants Convert Sunlight to Photocurrent through Different Pathways due to Different Architectures
Source: PLoS One. 2015 Apr 27;10(4):e0122616. doi: 10.1371/journal.pone.0122616 (PMC4411099; doi:10.1371/journal.pone.0122616)
Supplement: S3 Table — (DOCX) [file pone.0122616.s008.docx]

**Table S3. List of oligonucleotides used for the generation of the mutated tobacco lines.**

| Name | Sequence (5' – 3') |
| --- | --- |
| aadA-NdeI_Flast | ATCATATGGGGGAAGCGG |
| aadA-XbaI_R | GCTCTAGATTATTTGCCGACTACC |
| psbA-BamHI_F2 | ATGGATCCGTGCTTGGGAGTCCCTGA |
| rpl2-EagI_R1 | ATCGGCCGAACCGATATGCCCTTAGGCA |
| R238A_F | AATGAAGGTTACGCATTCGGTCAAGAGGAAGAAACTTATA |
| R238A_R | CTCTTGACCGAATGCGTAACCTTCATTAGCAGATTCATTTTC |
| R238D_F | GGTTACGATTTCGGTCAAGAGGAGGAAACTTATAACATCGTAGCCG |
| R238D_R | GTTTCCTCCTCTTGACCGAAATCGTAACCTTCATTAGCAGATTCATTTTC |
| R238E_F | AATGAAGGTTACGAATTCGGTCAAGAGGAAGAAACTTATA |
| R238E_R | CTCTTGACCGAATTCGTAACCTTCATTAGCAGATTCATTTTC |
